# Placental secretion of apolipoprotein A1 and E: the anti-atherogenic impact of the placenta

**DOI:** 10.1038/s41598-019-42522-1

**Published:** 2019-04-17

**Authors:** Hassan Melhem, Sampada Kallol, Xiao Huang, Michael Lüthi, Corneille Edgar Ontsouka, Adrian Keogh, Deborah Stroka, Wolfgang Thormann, Henning Schneider, Christiane Albrecht

**Affiliations:** 1 Institute of Biochemistry and Molecular Medicine, University of Bern, Bern, Switzerland; 2 Swiss National Centre of Competence in Research, NCCR TransCure, University of Bern, Bern, Switzerland; 3 Visceral Surgery and Medicine, Department for BioMedical Research, University of Bern, Bern, Switzerland; 4 Clinical Pharmacology Laboratory, Institute for Infectious Diseases, University of Bern, Bern, Switzerland; 5 Department of Obstetrics and Gynecology, University Hospital, University of Bern, Bern, Switzerland

**Keywords:** Reproductive biology, Molecular medicine

## Abstract

High levels of atherogenic lipids in pregnancy are associated with health complications for the mother, the fetus and the newborn. As endocrine secretory tissue, the human placenta releases apolipoproteins (apos), particularly apoA1 and apoE. However, the magnitude and the directionality of the apo secretions remain unknown. We aimed to 1) determine the amount and orientation (apical-maternal versus basal-fetal) of placentally secreted apoA1 and apoE using human perfused placenta and primary trophoblast cell (PTC) culture, 2) compare apoA1 and apoE secretions of PTC with that of hepatocytes and 3) associate the obtained results with human blood levels by determining apoA1 and apoE concentrations in maternal and fetal serum samples. In perfused placenta and serum samples, apoA1 and apoE concentrations were significantly higher at the maternal compared to the fetal side. For apoE a similar trend was found in PTC. For apoA1, the secretion to the apical side declined over time while release to the basal side was stable resulting in significantly different apoA1 concentrations between both sides. Unexpectedly, PTC secreted significantly higher amounts of apoA1 and apoE compared to hepatocytes. Our data indicate that the placenta may play an important role in maternal and fetal cholesterol homeostasis via secretion of anti-atherogenic apos.

## Introduction

Fetal development and growth depend on appropriate in utero homeostasis, which is regulated by placental transport and metabolism of maternal nutrients into the fetal circulation^1^. A dysregulation in the placental transport of nutrients is an important risk factor for complications during perinatal development that can extend into adult life^2,3^. In this context, cholesterol plays an important role as nutrient required for normal fetal development. Moreover, it is an essential component of cell membranes and for the synthesis of steroid hormones^4^. In the maternal lymphatic and blood circulations cholesterol is transported in the form of lipoproteins such as high-density lipoprotein (HDL), low-density lipoprotein (LDL) or very low-density lipoprotein (VLDL). During early developmental stages, when the fetus is incapable of its own cholesterol synthesis, placental transfer of maternal cholesterol is critical. Placental secretory and transport functions are regulated by numerous signals of fetal, maternal, and placental origin. Cholesterol is taken up at the maternal (apical) side of the placental syncytiotrophoblast (STB) through receptor-mediated endocytosis as well as receptor independent transport processes^5,6^. The implicated receptors are expressed in the placenta and/or STB^7,8^. To exit the fetal (basolateral) side of STB, cholesterol can be complexed with apolipoproteins (apos). Besides its beneficial aspects in human physiology, an excess of cholesterol can also be harmful^9^. Several observational studies consistently show an association between elevated LDL cholesterol and high prevalence of cardiovascular disease in adults, and, on the other hand, between high HDL cholesterol and reduced prevalence of these diseases^10–12^. Though this concept has been recently debated^10,13^, it is a well established and still widely accepted paradigm. Interestingly, pregnancy is a proatherogenic-like physiological state, which is accompanied by substantial dyslipidemia in second and third trimester^14,15^. Moreover, pathologies such as preeclampsia, a human pregnancy-related disease, impair maternal vascular functions and compromise fetal development. Indeed, preeclamptic mothers exhibit elevated total cholesterol, LDL-cholesterol and triglycerides in the blood^16–18^. If or to what extent the placenta does affect the pregnancy-related hyperlipidemia is currently not clear.

ApoA1 is a major apo of HDL cholesterol and is involved in the regulation of reverse cholesterol transport and the metabolism of HDL particles^19^. This transport process is regulated by a physical interaction of apoA1 with its molecular partner, the ATP-binding cassette (ABC) transporter A1 (ABCA1), located in the plasma membrane. In a very recent study, *Kallol et al*.^20^ demonstrated that ABCA1 is predominantly expressed on the apical (maternal) side of the placental STB. Similarly, efflux of cholesterol was detected mainly to the apical as compared to the basal (fetal) side of STB. Interestingly, the placental expression of ABCA1 is markedly reduced in preeclamptic pregnancies^21^.

ApoE, a major constituent of VLDL, has been shown to be an essential ligand for the uptake and clearance of atherogenic lipoproteins. Moreover, it has been demonstrated that apoE plays an important role in atherosclerosis by modifying inflammatory responses and facilitating cholesterol efflux from cells^22,23^. In support of these findings, genetic ablation of apoE was found to cause severe atherosclerosis throughout the arterial tree^24^. Additionally, it was suggested that apoE polymorphisms play an important role in the development of pre-eclampsia^25^ and apoE is crucial for fetal cholesterol metabolism^26^.

Studies performed in placental explants and in trophoblast cell cultures have shown that the placenta secretes apoB–containing lipoproteins^27^. Although the release of the two anti-atherogenic apos, apoA1 and apoE by the placenta has been reported^28,29^, the directionality of the secretion towards fetal, maternal or both compartments remains unknown. Additionally, the quantitative capacity of the placenta to synthesize, store and release apos has not been determined. These aspects, however, are fundamental as apos could have a major impact on the mother’s lipid profile during pregnancy and could play a supportive role in regulating maternal and fetal cholesterol homeostasis.

Therefore, the objectives of the present study were to (1) quantitatively assess placental apoA1 and apoE secretion, (2) determine the directionality (maternal versus fetal orientation) of apoA1 and apoE release by the human placenta, (3) compare apoA1 and apoE secretion in primary trophoblasts with that in hepatocytes and (4) analyze the physiological serum concentrations of apoA1 and apoE in corresponding maternal and fetal human serum samples. The quantity and directionality of apoA1 and apoE secretions were investigated by using two highly physiological models each of them offering maternal-like and fetal-like compartments: (1) the *ex vivo* dual perfusion of healthy term human placenta, and (2) an *in vitro* model with human trophoblast cell isolated from term placenta and cultured on Transwell filter supports forming a tight monolayer of STB. To quantitatively assess the amount of placental apo release, and thereby to determine the physiological relevance of placental apo secretion, we compared the secretion of apoA1 and apoE between primary trophoblasts and primary hepatocytes using conventional cell culture conditions. Indeed, the liver plays a key role in promoting physiological changes and adaptations occurring during pregnancy (for review, see^30^) and is one of the major organs involved in the regulation of lipid and lipoprotein homeostasis^31,32^. In the present work, we show for the first time that the placenta has the bi-directional capacity to secrete apoA1 and apoE with a predominant orientation to the apical (maternal) side. In line with the placenta perfusion data, apoA1 and apoE concentrations measured in corresponding maternal and fetal serum samples showed also significantly higher apoA1 and apoE levels in the mother. We further demonstrate that primary trophoblasts secrete higher levels of apoA1 and apoE compared to primary hepatocytes in culture. Collectively, these data indicate that the placenta plays an important role in the regulation of maternal and fetal cholesterol homeostasis via secretion of anti-atherogenic apos during pregnancy.

## Results

### *Ex vivo* dual perfusion model of the human placenta

#### Validation of the perfusion system

To determine the magnitude and the directionality of apoA1 and apoE secretion in a highly physiological system, we used the *ex vivo* dual perfusion model of the healthy human placenta. Depending on the size and area of the perfused cotyledon, a variable number of canulae were inserted at the maternal side. For the present experiments the weight of the of perfused cotyledons varied between 25.0 g and 49.6 g resulting in the insertion of 16 to 25 canulae. (Supplemental Table S2). To demonstrate integrity and viability of the perfused placental tissue several parameters including the volume of the fetal perfusate, pH, consumption of oxygen and glucose and production of lactate were monitored (Table 1). The pH in the maternal and fetal perfusates was within the physiological range of 7.3 to 7.4 and remained unchanged. The pO_2_ ranges for maternal arterial and venous samples were 133.0–147.7 mmHg and 76.4–109.0 mmHg, respectively (n = 4). In fetal arterial and venous samples the pO_2_ values ranged between 51.0–70.5 mmHg and 62.3–82.1 mmHg, respectively (n = 4). Lactate production was constant (0.33 ± 0.05 µmol/min/g after 60 and 240 minutes). Oxygen consumption was constant throughout perfusion (0.40 ± 0.10 µL/min/g after 60 and 240 minutes). Consumption of glucose showed a slight increase (0.122 ± 0.02 and 0.155 ± 0.122 µmol/min/g after 60 and 240 minutes, respectively). Clearance of antipyrine and creatinine were determined to assess matching between maternal and fetal circuit. Antipyrine clearance increased slightly (2.1 ± 0.4 and 2.5 ± 0.5 ml/min after 60 and 240 minutes, respectively) whereas creatinine clearance remained unchanged at 0.8 ± 0.1/0.2 ml/min (Table 1).

**Table 1:** Summary of placental perfusion parameters (n = 4).

Placental perfusion parameters							
	pH		O_2_ consumption μL/min/g	Glucose consumption μmol/min/g	Lactate production μmol/min/g	Antipyrine clearance ml/min	Creatinine clearance ml/min
	Maternal artery	Fetal artery					
60 minutes	7.4 ± 0.06	7.3 ± 0.01	0.4 ± 0.10	0.122 ± 0.02	0.33 ± 0.05	2.1 ± 0.4	0.8 ± 0.1
240 minutes	7.4 ± 0.04	7.3 ± 0.01	0.4 ± 0.10	0.155 ± 0.02	0.33 ± 0.05	2.5 ± 0.5	0.8 ± 0.2

#### Directionality of apolipoprotein secretion during perfusion

**Figure 1:**
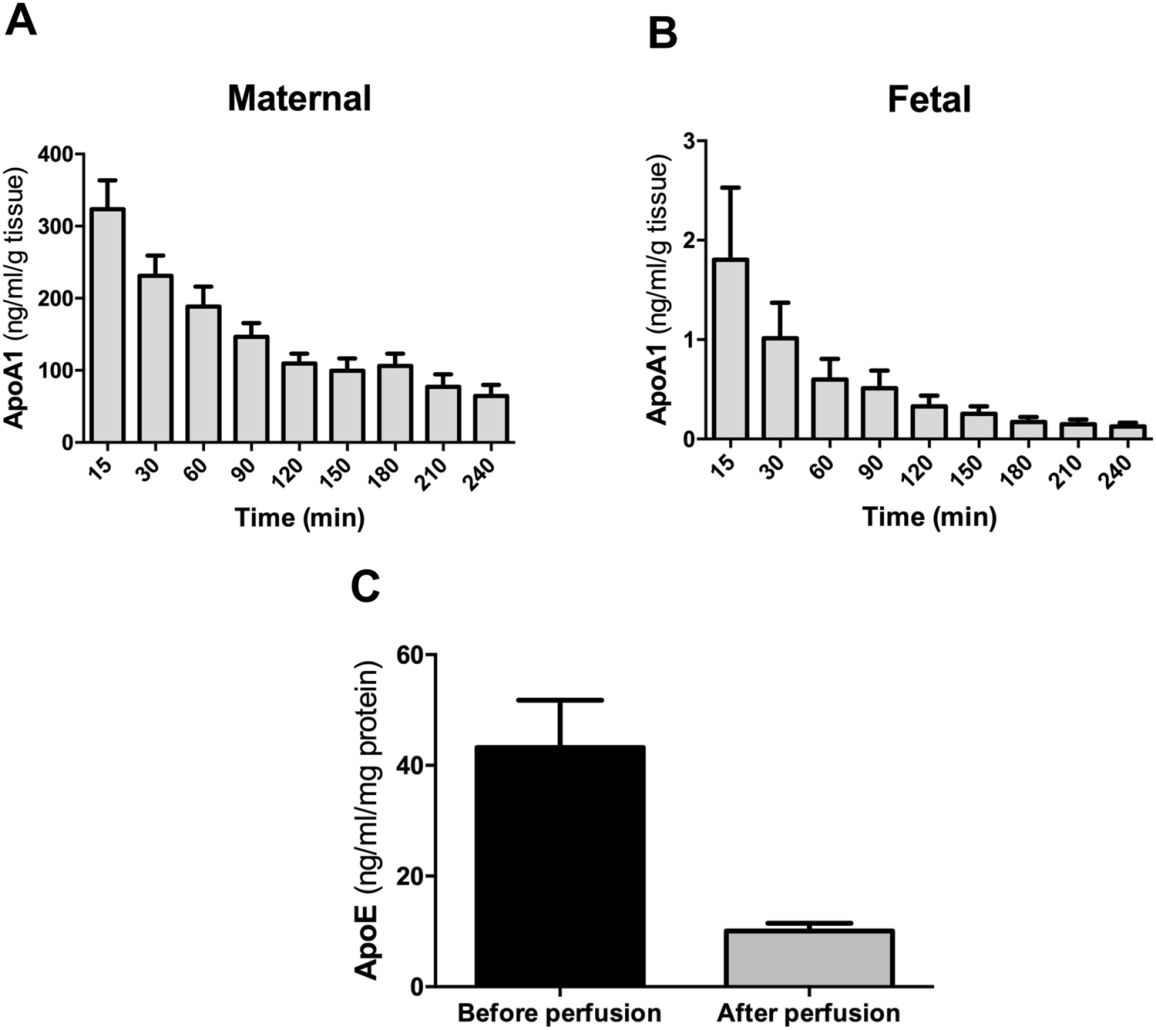
Secretion of apoA1 by the human perfused placenta. Four term human placentas were initially perfused for 30 minutes with DMEM medium and EBSS buffer containing 2 g/L of glucose, 10 g/L Dextran FP40, 40 g/L BSA and 2.5 IE/mL heparin. This washing phase was followed by 240 minutes perfusion of the maternal and fetal circulation as an open/open system. Samples were taken at 15, 30, 90, 120, 150, 180, 210 and 240 minutes. (**A**,**B**) The concentration of apoA1 in the maternal (**A**) and the fetal perfusate (**B**) was measured at each time point by ELISA and normalized to the weight of the cotyledon (g). (**C**) Measurement of apoA1 concentrations in placental tissue lysates before and after the perfusion by ELISA. The concentration was normalized to the total protein content (mg). Results represent mean ± SEM of four independent experiments measured in triplicates; *p < 0.05.

**Figure 2:**
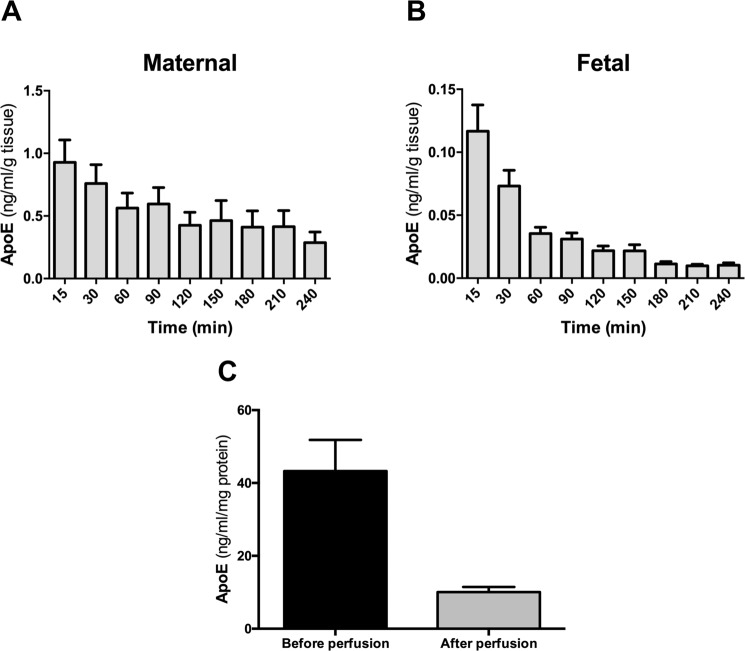
Secretion of apoE in the human perfused placenta. Four term human placentas were initially perfused for 30 minutes with DMEM medium and EBSS buffer containing 2 g/L of glucose, 10 g/L Dextran FP40, 40 g/L BSA and 2.5 IE/mL heparin. This washing phase was followed by 240 minutes perfusion of the maternal and fetal circulation as an open/open system. Samples were taken at 15, 30, 90, 120, 150, 180, 210 and 240 minutes. (**A**,**B**) The concentration of apoE in the maternal (**A**) and the fetal perfusate (**B**) was measured at each time point by ELISA and normalized to the weight of the cotyledon (g). (**C**) Measurement of apoE concentrations in placental tissue lysates before and after the perfusion by ELISA. The concentration was normalized to the total protein content (mg). Results represent mean ± SEM of four independent experiments measured in triplicates; *p < 0.05.

The measurement of apoA1 and apoE in the maternal and fetal perfusates revealed interesting secretion patterns. First of all, we observed that apoA1 and apoE were predominantly released to the maternal compartment (apoA1 ranging from 338.30 ± 41.31 ng/mL/g to 64.45 ± 15.52 ng/mL/g and apoE ranging from 1.34 ± 0.49 ng/mL/g to 0.12 ± 0.03 ng/mL/g) in comparison to the fetal compartment (apoA1 between 1.34 ± 0.49 ng/mL/g and 0.13 ± 0.04 ng/mL/g and apoE between 0.09 ± 0.01 ng/mL/g and 0.01 ± 0.001 ng/mL/g) (Figs 1 and 2A,B). To elucidate how the perfusion process alters the tissue content of the apos, we measured the concentrations of apoA1 and apoE in placental tissue before and after perfusion by ELISA. We found approximately 4-fold lower apoA1 and apoE concentrations in the placental tissues after four hours of perfusion as compared to adjacent non-perfused tissue (Figs 1C and 2C). During the course of perfusion, we noticed continuous decreases in apoA1 and apoE release towards both the maternal and fetal compartment (Figs 1 and 2, panels A and B). The release of apoA1 towards the maternal and fetal compartments was 4- and 14-times, respectively, lower at the end compared to the beginning of the perfusion. For apoE the corresponding decreases were 3- and 11-fold for the maternal and fetal side, respectively. In a next step, we evaluated whether the decrease in apo secretion is due to deteriorating placental function over perfusion time or if this is a consequence of diminishing placental apo content. Thus, we related the amount of the apos secreted at the beginning and at the end of perfusion to their corresponding stores at the respective time points (Figs 1C and 2C). When the data are expressed as the percentage of apo release related to the remaining availability within the tissue, we found that the secretion of apoA1 and apoE to the maternal compartment at the beginning and end of perfusion remained quite constant (11.60% vs 10.30%, for apoA1 and 2.14% vs 2.80%, for apoE, respectively). In contrast, the secretion of apoA1 and apoE to the fetal compartment was at the beginning about three fold higher than at the end of the perfusion when the remaining tissue content was taken into account (0.06 vs 0.02% for apoA and 0.27 vs 0.10% for apoE, respectively).

### *In vitro* primary trophoblast models

#### Directionality of apolipoprotein secretion in Transwell system

**Figure 3:**
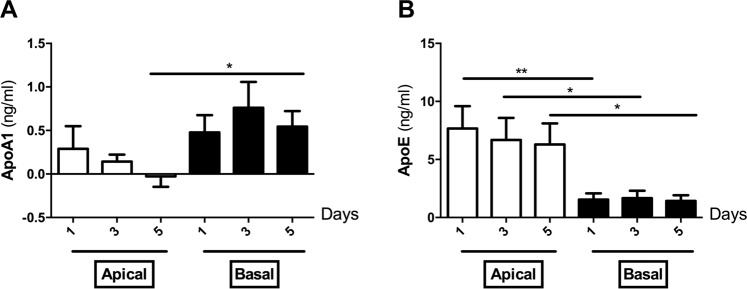
Secretion of apoA1 and apoE by primary trophoblasts seeded on Transwell filter supports. Primary trophoblast cells were isolated form healthy human term placentas (n = 5) and seeded at a density of  1× 10^6^ cells/cm^2^ on Transwell inserts. Cells were cultured for five days in DMEM containing 4.5 g/l glucose supplemented with 10% fetal bovine serum and antibiotic-antimycotic. Details of establishment of confluent human primary trophoblasts monolayer are described in the Materials and Methods section. The medium was collected at day one, three and five. (**A**,**B**) ELISA measurements of apoA1 (**A**) and apoE (**B**) concentrations in the apical and basal compartment. Results represent mean ± SEM of five independent experiments performed in triplicates; *p < 0.05; **p < 0.01.

In order to validate our findings from the placenta perfusion in an additional physiological system, we investigated the magnitude and directionality of apoA1 and apoE secretion in primary trophoblast cells grown as monolayers on Transwell filter supports. Overall, the secretion of apoA1 into the cell culture medium was approximately 10-fold lower than that of apoE (Fig. 3A and B). Although not statistically significant, the concentration of apoA1 showed a trend for a decrease during culturing time in the apical (maternal) chamber (0.28 ± 0.20 ng/mL, 0.14 ± 0.07 ng/mL and −0.02 ± 0.12 ng/mL for day 1, 3 and 5, respectively; Fig. 3A). No difference was found for apoA1 secretion in the basal (fetal) chamber during the culturing time (0.47 ± 1.90 ng/mL, 0.76 ± 0.29 ng/mL and 0.54 ± 0.17 ng/mL for day 1, 3 and 5, respectively; Fig. 3A). At day five, apoA1 secretion was significantly higher at the basal side in comparison to the apical compartment (−0.02 ± 0.12 ng/mL vs 0.54 ± 0.17 ng/mL; p = 0.0207; Fig. 3A). ApoE was continually secreted to both compartments and showed no decline over 5 days of culturing (Fig. 3B). However, at all-time points the secretion of apoE to the apical chamber (7.66 ± 1.90 ng/mL, 6.68 ± 1.89 ng/mL and 6.29 ± 1.80 ng/mL for day 1, 3 and 5, respectively) was significantly higher than to the basal chamber (1.54 ± 0.53 ng/mL, 1.67 ± 0.62 ng/mL and 1.42 ± 0.49 ng/mL for day 1, 3 and 5, respectively; Fig. 3B).

### Comparison of apo secretion between primary trophoblasts and primary hepatocytes

**Figure 4:**
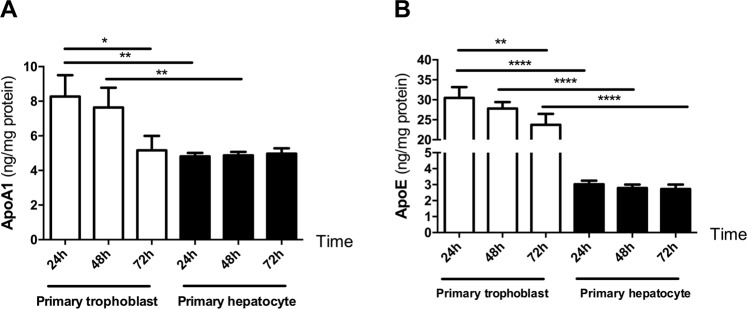
Secretion of apoA1 and apoE by primary hepatocytes and primary trophoblasts. Primary hepatocytes and primary trophoblast cells were isolated from human liver of colorectal metastases patients (n = 6) and from healthy human term placentas (n = 6) as described in the Materials and Methods section. Primary hepatocytes and primary trophoblasts were seeded at a density of 75000 cells/cm^2^ and 0.5 × 10^6^ cells/cm^2^, respectively, on conventional plates and cultured for three days. Each day, the medium was collected and cellular protein content determined. (**A**,**B**) Quantification of apoA1 (**A**) and apoE (**B**) by ELISA. The concentration was normalized to the respective protein concentration. Results represent mean ± SEM of six independent experiments performed in triplicates for primary hepatocytes and primary trophoblasts, respectively; **p < 0.01; ***p < 0.001.

In order to determine the potential physiological relevance of placental apo release, the secretory capacity of primary trophoblasts was compared to primary hepatocytes. After culturing cells on conventional plates for three days, apoA1 secretion was significantly higher (day 1: 8.26 ± 1.23 vs 4.81 ± 0.20 ng/mg protein; p = 0.005; day 2: 7.63 ± 1.1 vs 4.87 ± 0.19 ng/mg protein p = 0.007) in primary trophoblast as compared to primary hepatocytes (Fig. 4A). In agreement with our previous studies using primary trophoblast on the Transwell system (Fig. 3A), there was again a significant decrease in the secretion of apoA1 during the culturing time (day 1: 8.26 ± 1.23 vs day 3: 5.165 ± 0.83 ng/mg protein; p < 0.05; Fig. 4A). No difference in apoA1 secretion was observed in primary hepatocytes during three days of culture (Fig. 4A).

Similarly, apoE secretion was significantly higher (day 1: 30.48 ± 2.6 vs 3.02 ± 0.22 ng/mg protein; p < 0.0001; day 2: 27.80 ± 2.70 vs 2.79 ± 0.21 ng/mg protein; p < 0.0001; day 3: 23.70 ± 2.70 vs 2.72 ± 0.28 ng/mg protein; p < 0.0001) in primary trophoblasts as compared to primary hepatocytes (Fig. 4B). The amount of secreted apoE did not change during the culturing time in primary hepatocytes; however, there was a significant decrease of apoE secretion in primary trophoblast during the culturing time (day 1: 30.48 ± 2.6 vs day 3: 23.7 ± 2.7 ng/mg protein; p < 0.01; Fig. 4B).

### Comparison of apoA1 and apoE levels between maternal and fetal serum samples

**Figure 5:**
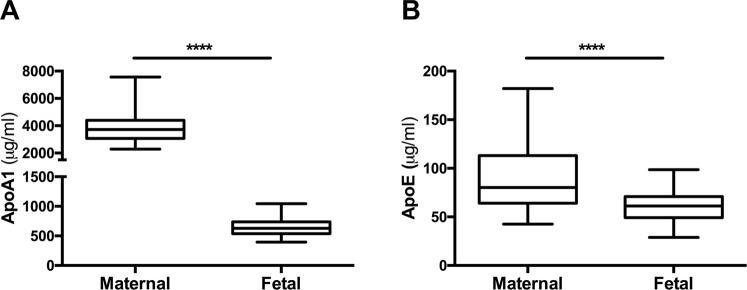
Quantification of apoA1 (**A**) and apoE (**B**) concentrations by ELISA in corresponding maternal and fetal serum samples collected immediately before (maternal) and within maximally one hour after delivery(fetal) by elective Cesarean section. Samples were obtained from healthy term pregnancies. Results represent mean ± SEM of 40 patients measured in duplicates; p < 0.0001.

To reinforce our *ex vivo* and *in vitro* data and relate them to the physiological context in the blood, we measured the levels of apoA1 and apoE in corresponding maternal and fetal serum samples (n = 40). ApoA1 concentrations were approximately 6-fold higher in maternal as compared to fetal serum samples (3874.0 ± 177.8 µg/ml vs 648.0 ± 23.2 µg/ml p < 0.0001, Fig. 5A). Similarly, a significant increase in the apoE levels was found in maternal as compared to the fetal serum samples (86.57 ± 4.90 µg/ml vs 60.33 ± 2.50 µg/ml p < 0.0001, Fig. 5B). However, the difference between maternal and fetal apoE serum levels was less pronounced (20% increase) as compared to apoA1 (600% increase).

## Discussion

In the present work, we determined for the first time the magnitude as well as the directionality of placental apoA1 and apoE secretion in two highly physiological human placenta models. We found that human term placenta is able to secrete apoA1 and apoE to both the maternal and the fetal side. While apoA1 secretion in the human placenta perfusion model was predominantly orientated towards the maternal side, the Transwell system with primary trophoblast cells suggested higher apoA1 release towards the fetal side. For apoE, however, the secretion pattern consistently showed a predominant maternal orientation in both placenta models. Increased apoA1 and apoE concentrations at the maternal compartment were also found when corresponding maternal and fetal serum samples were analyzed. The magnitude of placental apo release was assessed by comparing the apo secretion capacities of human primary trophoblasts with human primary hepatocytes. We demonstrated that primary trophoblasts secrete comparable or even higher amounts of apoA1 and apoE than primary hepatocytes.

In order to validate the placenta perfusion system and to guarantee the reproducibility of the experiments, several parameters (e.g. pH, oxygen, carbon dioxide, glucose, lactate) were analyzed in the perfusates. Consistent with previous reports^33,34^, only perfusions with a leak of maximum 10% of the initial volume of fetal medium were included in this study. The measured parameters confirmed the reliability and reproducibility of the perfusions. Indeed, throughout all perfusion experiments, pH in the maternal and fetal perfusate were within the physiological range of 7.3 to 7.4 and remained unchanged. The consumption of oxygen and glucose as well as lactate production, indicators of the metabolic condition of the perfused tissue, are also in line with previous reports^34–36^. Clearance of antipyrine and creatinine, molecules that passively pass membranes, are commonly used to provide reliable information on the overlap of the maternal and fetal circuits. In the present study, antipyrine and creatinine clearance are in accordance with published results of other laboratories^37^.

Using this validated *ex vivo* system, we aimed to determine the role of the placenta in the secretion of antiatherogenic lipoproteins. Pregnancy is characterized by important physiological changes in the maternal organism resulting in hyperlipidemia^30^, exposing the fetus to increased amounts of lipids. It has been reported that uptake of cholesterol by trophoblasts occurs via interaction of HDL and LDL/VLDL with lipoprotein receptors present at the apical trophoblast plasma membrane^5^. However, the route by which cholesterol exits the basal trophoblast membrane remains only poorly characterized. It has been demonstrated that cholesterol exits the trophoblast by secretion in newly formed lipoproteins or by efflux to acceptor molecules^3,38,39^. Studies reported that human term placental biopsies secrete apoB100-containing lipoproteins^27^, and that the choriocarcinoma cell line BeWo is able to release apoB^40^. To the best of our knowledge, only two reports have demonstrated secretion of apoA1 and apoE by the human placenta^28,29^. The results of our *ex vivo* studies indicate that human term placenta secretes apoA1 and apoE to both the maternal and the fetal side. The release of apoA1 and apoE by the human term placenta, however, is predominantly directed towards the mother. In the present study both the secretion and the remaining stores of apoA1 and apoE in the placenta declined throughout the perfusion period. However, important differences were detected between the secretion patterns into the maternal and the fetal side. Indeed, the secretion of apoA1 and apoE to the maternal compartment seemed to be dependent on the placental stores. This was concluded from the finding that the apo fraction released towards the maternal side correlated well with the remaining apo concentration in the placental tissue. In contrast, when relating the apo secretion to the remaining placental tissue content at the fetal compartment, the secreted fraction of apoA1 and apoE was at the beginning approximately three fold higher than at the end of the perfusion. Taken together, these data suggest that with decreasing placental apoA1 and apoE stores, the “priority” of the apoA1 and apoE release by the placenta is directed towards the mother.

To reinforce our *ex vivo* and *in vitro* data and relate them to the physiological context in the blood, we measured the levels of apoA1 and apoE in corresponding maternal and fetal serum samples obtained from healthy pregnancies. ApoA1 concentrations were significantly (600%) higher in maternal as compared to fetal serum samples. A similar and significant, but markedly less pronounced gradient (20%) was found for apoE. These findings are in line with previous findings from Mistry *et al*. showing higher concentrations of apoA1 and apoE in the maternal as compared to the fetal plasma^41^. Interestingly, the dimension of the apo gradients between maternal and fetal serum samples measured in our cohort corresponds well with the results of the placenta perfusion studies. Thus, when calculating the ratio between maternal and fetal release in the placenta perfusion experiments, a much higher predominance for the maternal side was found for apoA1. Taken together these data suggest that the placenta plays a major role in regulating the apolipoprotein levels of the pregnant mother and the fetus. The findings of the present study are also consistent with the hypothesis that increased levels of antiatherogenic apoA1 and apoE in the maternal blood may protect the mother from health complications arising from high lipid levels during pregnancy.

The physiological implications of the predominant maternally orientated secretion pattern found in the present study are still unclear. However, previous studies showed that ABCA1, the molecular partner interacting with apoA1 to mediate cholesterol transport, is abundantly expressed at the maternal side in term placenta^20,42^. Thus, ABCA1-mediated cholesterol efflux may be supported *in situ* by placental release of apoA1. Moreover, it is tempting to speculate that the placenta contributes to the regulation of the mother’s lipid profile during pregnancy by secreting antiatherogenic apos. During pregnancy an increase of maternal lipid levels including triglycerides and total cholesterol has been reported^15,43^ and was associated with serious consequences for both the mother and the child, such as pregnancy-induced hypertension^44,45^, preeclampsia^16,46^ and preterm birth^47,48^.

To provide additional evidence for the apical or basal secretion of apos and to determine the cellular origin of apo release, primary trophoblasts growing on Transwell permeable filter support were used. This *in vitro* model has been previously used to study trophoblast transport from the apical (maternal) to the basal (fetal) side^49,50^. Using this system, we confirmed that cultured trophoblasts secrete apoE predominantly to the apical compartment. Secretion to the basal side was detectable, but approximately four times lower. The remarkably higher secretion of apoA1 to the maternal side found in the placenta perfusion studies, could, however, not be confirmed by the primary trophoblast experiments. This may be associated with the fact that apical apoA1 secretion decreased over time in culture, and was already negligible when a tight monolayer was formed. The constant decline in apoA1 secretion in trophoblasts may be associated with the continuous exposure of these cells to high (25 mM) glucose levels in the culture medium. A high glucose concentration in the medium is a technical prerequisite for proper adhesion and growth of primary trophoblast cells, but was also shown to inhibit apoA1 synthesis in epithelial cell types such as HepG2 cells^51^. In contrast, apoE secretion seems to be not majorly affected by high glucose levels as demonstrated in rats fed by a high glucose diet^52^. Interestingly, the basal secretion of apoA1 was maintained during the monolayer formation. This phenomenon could be related to the technical setup of the Transwell system: the apical surface of the monolayer is in direct and constant contact with high glucose containing medium while at the basal side this condition is to a certain degree attenuated by the presence of a PET membrane coated with Matrigel.

On the other side, it is also possible that specific components present in the blood are needed for continuous secretion of apoA1, which were lacking in our cell culture system. This has been shown for apoB secretion which increased at the apical side only after treatment with estrogen^53^. Additionally, it has been demonstrated that the mRNA expression level of apoA1 was induced in response to glucocorticoids and insulin in primary hepatocyte cells^54^. However, there are also indications that the stimulating effects of certain hormones (e.g. growth hormone, cortisol or thyroxine), which can affect metabolism and release of apos (e.g. apoB48 and apoB100), depend on whether they act on intact organs or isolated cells^55^. Moreover, the fact that our experiments were performed in the absence of cholesterol, could be a factor influencing the release and directionality of apos. In this context it has been previously shown that the cellular lipid content influences the synthesis and secretion of apoA1^6,56^ and plasma HDL can affect the stability and secretion of apoA1^57–59^. Thus future experiments in polarized cell and perfusion models are warranted to investigate the contribution of cholesterol in affecting apo release and stabilization.

An alternative explanation for the discrepancy between the apoA1 results of the perfusion and the trophoblast Transwell model is that the latter lacks the contributions from stroma and endothelium. The perfused cotyledon comprises different cell populations like stroma and endothelium which may play a role in the secretion of apoA1. Although studies on the role of placental endothelial and stromal cells in apo homeostasis during pregnancy are scarce, synthesis of apoA1 and apoB by rabbit and bovine endothelium have been reported^60,61^. Similarly, the placental stroma tissue is often neglected in transport studies. Since the Glut4 transporter was unexpectedly shown to be expressed in villous stromal cells^62^, it became clear that the understanding of materno-fetal nutrient transport and related secretion processes becomes more complex. Hence in future work, it is important to take the endothelial and stromal cells into consideration. In this context, the future establishment of a syncytium-endothelium co-culture model with primary cells could be beneficial for further clarification of apo homeostasis during pregnancy.

The fact the placenta secretes a considerable amount of apoA1 and apoE preferentially to the maternal side, prompted us to further investigate the physiological effects and consequences of this directional release. As apoA1 is an important acceptor protein for cholesterol and plays a crucial role in reverse cholesterol transport, we hypothesized that cholesterol efflux from term placenta occurs predominantly to the mother. Consistently, cholesterol efflux experiments performed in primary trophoblasts grown to confluent monolayers showed a significantly higher cholesterol transport to the apical (maternal) as compared to the basal (fetal) side, independent of the addition of exogenous apoA1. These results are of physiological importance since cholesterol accumulation in the placenta is considered as one of the major causes for pregnancy complications and diseases for the fetus^63,64^. Of note, the current experiments were performed exclusively on term placenta, when the fetus is rather independent from the maternal cholesterol supply. These observations suggest that at term the placenta may exert a protective role both for the mother and the fetus by secreting anti-atherogenic apos. The latter have not only beneficial effects for the mother’s lipid homeostasis, thereby decreasing her risk to develop gestational complications (e.g. preeclampsia), but may also protect the fetus, through increasing cholesterol efflux to the maternal side.

The findings reported in the current study suggest that the placenta is an additional important organ capable of producing high levels of apos, especially apoA1 and apoE. Indeed, we found that apoA1 and apoE secretion by primary trophoblasts was equal or even higher than that observed from primary hepatocytes. Primary hepatocytes are known to secrete high amounts of apos^31,32,65^ and thus served as a well-accepted model of epithelial cells which have a proven role in regulating lipid homeostasis. The high amounts of apoA1 and apoE secreted by placental trophoblast cells in comparison with hepatocytes argues for a considerable contribution of the placenta in mediating during pregnancy the well documented anti-atherogenic effects of apoA1 and apoE^22,24,66^. These findings further highlight a yet underestimated physiological role of the placenta, as currently only the liver and the intestine are considered as the predominant sources of apo secretion with major impact on lipid homeostasis^31,32^.

In summary, our results provide evidence for apical and basal secretion of two antiatherogenic apos: apoA1 and apoE. Interestingly, the secretion of these two apos occurs predominantly to the apical (maternal) side which is in agreement with higher serum concentrations of both apos measured in the mother. Additionally, the comparison between hepatocytes and primary trophoblasts in culture confirmed that placental trophoblast cells secrete similar or even higher amounts of apoA1 and apoE than hepatocytes. Taken together, these data imply that in term pregnancies the predominantly apical release of apoA1 and apoE is relevant for the regulation of maternal cholesterol homeostasis.

## Materials and Methods

### Placenta collection

Human placental tissues were collected from the Division of Obstetrics and Gynecology, Lindenhofgruppe Bern, Switzerland, under approval by the ethical commission of the Canton of Bern (approval No Basec. 2016-00250). Written informed consent was obtained from all participants. Placentas were collected from uncomplicated pregnancies following elective cesarean section beyond 37 weeks of gestation without prior labour upon patients request or due to breech presentation. Table 2 shows a summary of the subject characteristics. All experiments were carried out in accordance with the relevant guidelines and regulations.

**Table 2:** Clinical parameters of maternal, placental and fetal subjects (n = 15).

Clinical data							
Mother							
						Blood pressure (mmHg)	
Age (years)	Weight at birth (kg)	Height (cm)	BMI at birth (kg/m^2^)	Gestational age (weeks)	Smoker	Systolic	Diastolic
34.4 ± 1.1	78.53 ± 4.9	167.1 ± 2.2	27.8 ± 1.2	37.73 ± 0.3	0/12	118.1 ± 3.6	72.4 ± 2.8
**Placenta**				**Newborn**			
**Weight (g)**		**Umbilical cord length (cm)**		**Gender**		**Weight (g)**	
618.9 ± 26.6		15.80 ± 1.9		9♂, 6♀		3051 ± 128.1	

### Placenta perfusion

For *ex vivo* studies, the single cotyledon placenta perfusion system was used as described by Schneider *et al*.^67^. Briefly, a fetal artery and vein on the chorionic plate were cannulated to set up a vascular circuit. Perfusion was started using Dulbecco’s modified Eagle medium (DMEM) and Earle’s balanced salt solution (EBSS) containing 2 g/L of glucose (Sigma, USA), 10 g/L Dextran FP40 (Serva, Germany) and 40 g/L bovine serum albumin (BSA, Sigma, USA). After confirmation of circulatory integrity, the cotyledon and portion of its surrounding placental tissue were transferred to a temperature-controlled (37 °C) chamber, where perfusion of the fetal circulation was continued as an open system at a flow rate of 15 mL/hour/cannula. The cylinder gas composition for the two circulations was 19% O_2_/5% CO_2_/76% N_2_ at the maternal and 5% CO_2_/95% N_2_ at the fetal side, respectively.

The original method of Schneider *et al*.^67^ had been modified by Soydemir *et al*.^68^, who instead of three to five cannulae used 22 cannulae for the maternal circuit. The cannulae were inserted at alternating depths of 1 and 2 cm into the intervillous space. This modification allowed a better distribution of maternal perfusate inside the intervillous space and improved oxygenation of the trophoblast was reflected by the secreted cytokine profile^69^. For the present study depending on the cotyledon size 5 to 25 cannulae were inserted into the intervillous space to establish the maternal circulation. Maternal flow rate was initiated at 30 mL/hour/cannula as an open system.

The maternal and fetal artery pressure was monitored by a Millar instrument (Millar instruments, USA). The placental cotyledon circulations that leaked secondary to lack of vascular integrity or that did not achieve a stable pressure were excluded. After an initial 30 minutes washing phase, maternal and fetal venous perfusate samples were taken at 15, 30, 60, 120, 150, 180, 210 and 240 minutes (n = 4). Samples were collected in triplicate and centrifuged at 1100 g for 10 min at 4 °C to remove potentially eluted residual blood. Maternal and fetal perfusate samples were stored at −80 °C for subsequent analysis of apoA and apoE as described below.

Several parameters including the volume of the fetal perfusate, pH, oxygen consumption, glucose consumption, lactate production, antipyrine and creatinine transfer^34^ were evaluated in order to validate the perfusion system (Table 1). The antipyrine and creatinine concentrations in the placenta perfusion medium were 80 and 150 µg/mL, respectively. The formulas used for the calculation of these parameters are shown in Supplemental Table S1.

### Measurement of antipyrine and creatinine by capillary electrophoresis

Antipyrine and creatinine in the perfusate were monitored with a laboratory developed method based on micellar electrokinetic capillary chromatography (MECC). The assay comprises protein precipitation with trichloroacetic acid (TCA), hydrodynamic injection of alkalinized sample and analysis at pH 9.3 in a phosphate/tetraborate MECC buffer with 50 mM SDS and 2% methanol. The buffer system is similar to that used previously for drug monitoring of antipyrine in plasma^70^. Calibrators, controls and blank sample were prepared with perfusion medium that contained 2.5 units/mL liquemine (sodium heparin) and all reagents used were of analytical grade. 100 µL of perfusate, 20 µL of water, 20 µL of internal standard solution (400 µg/mL caffeine, solution stored at −20 °C) and 40 µL of 0.5 M TCA solution were pipetted into a 0.5 mL Eppendorf vial, vortexed for 30 s and centrifuged at 13000 rpm for 5 min. 120 µL of the supernatant was transferred into a 0.5 mL plastic tube (Semadeni, Ostermundigen, Switzerland) and alkalinized with 20 µL of 0.5 M NaOH. A P/ACE MDQ instrument (Beckman Coulter, Fullerton, CA, USA) equipped with a 50 µm I.D. fused-silica capillary (Polymicro Technologies, Phoenix, AZ, USA) of 60 cm total length (effective length 50 cm) was used. Samples were injected hydrodynamically via applying 1 psi (1 psi = 6894.8 Pa) for 6 s. A voltage of 21 kV (normal polarity) was applied. The current was about 28 µA. Sample storage and capillary cartridge temperatures were set to 20 and 22 °C, respectively, and analyte detection was effected at 200 nm (PDA detector). Creatinine, caffeine (internal standard) and antipyrine were detected after 5.6, 7.9 and 10.1 min, respectively. Quantification was based on seven-level internal calibration (antipyrine: 1–100 µg/mL; creatinine: 2–200 µg/mL) and the lowest calibrators were used as quantification limits. Interday precision was <5.0% for all compounds.

### Primary trophoblast isolation and characterization

For *in vitro* studies, we isolated villous trophoblasts from healthy human term placentas by enzymatic digestion and gravitational separation as previously described by Nikitina *et al*.^71^ with minor modifications. Briefly, villi-rich tissues were digested four times with 0.25% trypsin (Sigma, USA) and 300 IU/ml Deoxyribonuclease I (Sigma, USA), and then subjected to Percoll^®^ (Sigma, USA) density gradient centrifugation to isolate villous CTB. In order to assure the purity of the cells, flow cytometry analysis was performed by using the trophoblast-specific epithelial cell marker cytokeratin 7^72^ (anti-cytokeratin 7, Dako, Switzerland). Vimentin (anti-vimentin, Sigma, USA), which is only expressed in potentially contaminating cells (e.g. mesenchymal cells, fibroblasts, smooth muscle cells, stromal cells)^73^ served as a negative marker.

### Primary trophoblast cell culture

#### Conventional culture plates

Isolated human trophoblast cells were cultured in Dulbecco’s modified Eagle’s medium containing 4.5 g/l glucose (DMEM-HG, Gibco, UK) supplemented with 10% fetal bovine serum (FBS, Seraglob, Switzerland) and antibiotic-antimycotic (Gibco, USA) in a humidified incubator under a 5% CO_2_ atmosphere at 37 °C. Cells were seeded at a density of 0.5 × 10^6^ cells/cm^2^ in costar CellBIND– 24 well plates (Corning, USA). The morphology of the cells was monitored with a Leica DMi1 microscope (Leica, Germany) and the medium was collected daily for 3 days to measure apo release by enzyme-linked immunosorbent assay (ELISA; see below).

#### Transwell experiments with primary trophoblasts

To determine the directionality of apoA1 and apoE release, we used trophoblast confluent monolayer grown on Transwell inserts (Falcon, USA) composed of apical (maternal) and basal (fetal) compartments. The trophoblasts were seeded at a density of 1 × 10^6^ cells/cm^2^ on 6 well or 12 well Transwell inserts with 0.4 μm pore polyester membrane which were coated with Matrigel (BD Biosciences, USA) at a concentration of 25 µg/cm^2^ prior to the experiment. The trophoblast monolayer formation was evaluated by the tightness and barrier integrity as described^74^. In brief, trans-epithelial electrical resistance (TEER) was measured every day for 5 days using Millicell ERS-2 Volt-Ohm Meter (Millipore, USA) as described^74^. The TEER value was determined on the Matrigel-coated inserts in the presence or absence of trophoblast cells. Additionally, the vectorial permeability properties were evaluated by the diffusion of fluorescent dye Lucifer Yellow (Sigma, USA) as described in^74^. In the applied Transwell cell model, the human villous trophoblast barrier represents a continuous STB layer together with underlying CTB, proven by electron microscopy, mRNA and protein expressions of specific markers^74^. Stacked multinucleated and mononucleated cells were observed using electron microscopy; staining with the CTB marker E-cadherin revealed predominant expression in the mononuclear cells.

After confirmation of monolayer formation medium samples were collected for the measurement of apoA1 and apoE secretion from the apical and basal compartments on day 1, 3 and 5 of culture.

### Primary hepatocyte isolation and cultivation

Normal human liver tissue was taken from the periphery of liver specimens from patients undergoing surgical resection for colorectal metastases. Informed consent of the patients was obtained in accordance with institutional guidelines and the local ethics committee. The isolation procedure is described in detail by Portmann *et al*.^75^ in the Supplementary Information. Briefly, cells were isolated using a two-step enzymatic perfusion protocol. The viability of the isolated hepatocytes was determined by trypan blue exclusion, and only preparations of over 90% viability were used. The hepatocytes were seeded at a density of 75’000 cells/cm^2^ onto rat tail collagen coated 6 well tissue culture plastics in DMEM containing 10% fetal bovine serum (FBS) (Life Technologies, Switzerland), left to attach for 1 to 2 hours, and then washed twice with phosphate-buffered saline (PBS) (Life Technologies, Switzerland) to remove unattached cells. The hepatocytes were cultured in arginine-free medium made from a powdered base according to the formulation of Williams-E, supplemented with insulin (0.015 IU/mL, NovaRapid, Novo Nordisk), hydrocortisone (5 μmol/L, Sigma, Switzerland), penicillin, streptomycin, glutamine (100 IU/mL, 100 μg/mL, 2 mmol/L, GPS, Life Technologies, Switzerland), and ornithine (0.4 mmol/L, Sigma, Switzerland). During 3 days in culture, medium was collected daily and analyzed for apoA and apoE secretion.

The quality and reproducibility of hepatocytes produced using this method is described in Bhogal *et al*.^76^. Herein the authors refer to “normal” liver when the hepatocytes are isolated from resections which is the same source we used for the current experiments.

### Collection of blood samples

Blood from healthy pregnant women was collected from the maternal vein before delivery (n = 40) and from the umbilical cord vein immediately after delivery of the placenta (n = 40). Blood samples were collected in S-Monovette serum tubes (Sarstedt AG, Germany) and processed within 1 h. Samples were allowed to clot at RT and then serum was obtained after centrifugation at 2000 g for 10 mins at 4 °C. Serum samples were aliquoted and stored at −20 °C until further use as described in^77,78^.

### ELISA analysis of apoA1 and apoE secretion

ApoA1 and apoE were measured by a commercially available ELISA kit (Mabtech AB, Germany) according to the manufacturer’s instructions. In brief, the range of the standard curve was from 0.1 to 1000 ng/ml and from 0.02 to 100 ng/ml for apoA1 and apoE, respectively. The perfusion samples of the maternal side were diluted 1/300 and 1/5 for apoA1 and apoE, respectively, using Dulbecco’s phosphate buffered saline (DPBS, Sigma, USA) with 0.05% Tween-20 (Sigma, USA) containing 0.1% bovine serum albumin (BSA, Sigma, USA). The serum samples were diluted 1/200’000 for apoA1 and 1/20’000 for apoE in Apo ELISA buffer as suggested in the manufacturer’s instructions. 100 or 200 μl of standard and diluted samples were dispensed into wells. After two-hour incubation, the samples were aspirated and the wells were washed five times with DPBS containing 0.05% Tween-20. The antibody-HRP conjugate was then applied for one hour. After five consecutive washing steps, the substrate solution p-nitrophenyl-phosphate (pNPP, Sigma, USA) was added and left for one hour until completion of the chemical reaction. The absorbance was measured at 405 nm using a Vmax4.8 microplate reader (Molecular Devices, USA). The concentrations were calculated by subtracting the mean absorbance value of the blank from the standard and sample values prior to creating the standard curve. The concentrations of apoA1 and apoE secretion in samples were then interpolated from the slope of the respective standard curve and the obtained values were multiplied with the dilution factor. Finally, the concentrations of apo release were normalized to the weight of the cotyledon (perfusion samples) or to the protein concentration (tissue and cell samples). Placental tissue lysates before and after the perfusion were prepared using hypotonic buffer supplemented with protease inhibitors. Protein concentrations in placental tissues and cells were measured using Pierce BCA protein assay kit (Thermo Scientific, USA).

### Statistics

All statistical analyses were performed with GraphPad Prism software (GraphPad software, USA). Data are shown as mean ± SEM of at least four independent experiments performed in triplicates. The changes in apoA-1 and apoE secretion levels in *ex-vivo* and *in vitro* models were tested for significance by using one-way ANOVA followed by Tukey’s post-test. The apo contents in placental tissues before and after perfusion as well as the maternal and fetal serum samples were tested by using paired t-test. The alpha level of all mentioned tests was 0.05. The level of statistical significance was set at p < 0.05.

## Supplementary information

Supplementary tables 1 and 2

## Data Availability

The datasets generated during and/or analysed during the current study are available from the corresponding author on reasonable request.
