# Supplementary material for: Placental secretion of apolipoprotein A1 and E: the anti-atherogenic impact of the placenta
Source: Sci Rep. 2019 Apr 17;9:6225. doi: 10.1038/s41598-019-42522-1 (PMC6470155; doi:10.1038/s41598-019-42522-1)
Supplement: Supplementary file 1 — Supplementary tables 1 and 2 [file 41598_2019_42522_MOESM1_ESM.docx]

### Placental secretion of apolipoprotein A1 and E: the anti-atherogenic impact of the placenta

### Hassan Melhem^a,b1^, Sampada Kallol^a,b1^, Xiao Huang^a,b^, Michael Lüthi^a,b^, Corneille Edgar Ontsouka^a,b^ Adrian Keogh^c^, Deborah Stroka^c^, Wolfgang Thormann^d^, Henning Schneider^e^, and Christiane Albrecht*^a,b^Ung, s

### ^a^ [Institute of Biochemistry and Molecular Medicine, University of Bern](http://www.ibmm.unibe.ch/), Switzerland; ^b^ Swiss National Centre of Competence in Research, NCCR TransCure, University of Bern, Switzerland; ^c^ Visceral Surgery and Medicine, Department for BioMedical Research, University of Bern, Switzerland; ^d^ Clinical Pharmacology Laboratory, Institute for Infectious Diseases, University of Bern, Switzerland; ^e^ Department of Obstetrics and Gynecology, University Hospital, University of Bern, Switzerland

### ^1^Equal contribution

***Corresponding author**

Prof. Christiane Albrecht

University of Bern

Institute of Biochemistry and Molecular Medicine,

Bühlstrasse 28

CH-3012 Bern, Switzerland

Phone: +41-31-631 4108

Fax: +41-31-631 3737

E-mail: christiane.albrecht@ibmm.unibe.ch

**Supplemental Table S1:** Formulas for calculation of placenta perfusion parameters

* α represents the coefficient for physical solubility of oxygen in buffer (0.0239 ml/ ml buffer; see Cotes et. al., 2009)

pB = barometric pressure; mean pB = 693.5 mmHg

pH_2_O = partial pressure of water; mean pH_2_O = 47 mmHg

**Ref**.: Cotes, J. E., Chinn, D. J. and Miller, M. R. (2009). Transfer of Gases into Blood in Alveolar Capillaries. In Lung Function (eds J. E. Cotes, D. J. Chinn and M. R. Miller). doi:[10.1002/9781444312829.ch19](https://doi.org/10.1002/9781444312829.ch19)

**Supplemental Table S2:** Cotyledon weights and number of canulae used for placental perfusions

| No. of placenta perfusion | Cotyledon weight (g) | No. of canulae |
| --- | --- | --- |
| 1 | 35.28 | 18 |
| 2 | 24.99 | 16 |
| 3 | 49.63 | 25 |
| 4 | 26.65 | 25 |
